# PolyC-RNA-binding protein 1 (PCBP1) enhances tropomyosin 3 (TPM3) mRNA stability to promote the progression of esophageal squamous cell carcinoma

**DOI:** 10.1080/21655979.2022.2053801

**Published:** 2022-03-24

**Authors:** Kaiming Peng, Xiaoqiang Chen, Anqin Lin, Zhangwei Tong, Wenwei Lin

**Affiliations:** a Department of Thoracic Surgery, Fujian Medical University Union Hospital, Fuzhou, China; b Department of Otolaryngology, Fujian Medical University Union Hospital, Fuzhou, China; c Department of Surgery, Fujian Medical University Union Hospital, Fuzhou, China

**Keywords:** PCBP1, TPM3, mRNA stability, esophageal squamous cell carcinoma (ESCC)

## Abstract

The molecular etiology of esophageal squamous cell carcinoma (ESCC) has not been fully elucidated. Understanding the molecular mechanisms and finding new therapeutic targets for ESCC are of crucial importance. PolyC-RNA-binding protein 1 (PCBP1) is an RNA-binding protein. Here, we found overexpressed PCBP1 in esophageal cancer tissues by quantitative polymerase chain reaction (qPCR) and western blotting analysis. PCBP1 knockdown significantly attenuated migratory and invasion abilities of ESCC cells. Mechanistically, PCBP1 bound directly to tropomyosin 3 (TPM3) mRNA, which was verified by RNA–protein immunoprecipitation (RIP) assay. PCBP1 knockdown markedly reduced messenger RNA (mRNA) levels of TPM3. After inhibiting intracellular mRNA synthesis with actinomycin D (ActD), it was found that PCBP1 knockdown contributed to a significant decrease in TPM3 mRNA degradation. Furthermore, PCBP1 promoted migration and invasion of EC cells by directly binding to the 3’UTR of TPM3 mRNA, increasing TPM3 mRNA stability. Taken together, PCBP1 acting as a pro-oncogenic factor enhances TPM3 mRNA stability by directly binding to the 3’UTR of TPM3 mRNA in esophageal squamous cell carcinoma. Our findings provide a new perspective for understanding the molecular mechanism of esophageal carcinogenesis, and PCBP1 is a promising therapeutic target.

## Graphical abstract

**Figure:**
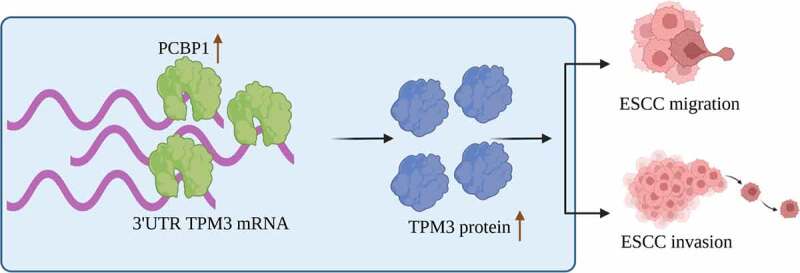

## Introduction

Esophageal cancer (EC) is a great challenge to human health, with 600,000 patients diagnosed and 540,000 deaths each year globally [1]. Esophageal squamous cell carcinoma (ESCC) is the main pathological characteristic of EC [2], which arises from malignant transformed esophageal epithelial cells [3]. Most cases of EC are diagnosed at an advanced stage, and the 5-year overall survival is less than 20% [4].

Extensive studies have explored the pathogenesis of esophageal cancer. Dietary habits contribute to the high incidence of esophageal cancer in China. Previous studies have reported that a high consumption of pickled foods significantly elevated the risk of esophageal cancer [^5–7^]. And drinking hot tea caused persistent damage to the esophageal epithelium, resulting in tumorigenesis [8]. Besides, *Porphyromonas gingivalis* facilitates esophageal squamous cell cancer progression by mediating TGFβ-dependent Smad/YAP/TAZ signaling pathway [9] and the miR-194/GRHL3/PTEN/Akt signaling axis [10]. Antibacterial therapies reduced the incidence of esophageal cancer [11,12]. Additionally, genetic factors play an important role in esophageal cancer development and progression [13,14]. The pathogenesis of esophageal cancer metastasis is complex. Therefore, further investigation may provide more insight into the precise pathogenesis of ESCC.

Tropomyosin 3 (TPM3) belongs to the TPM family that can maintain the stabilization of cytoskeletal microfilaments [15,16]. Our previous work showed that TPM3 expression was increased in ESCC tissues and high expression of TPM3 was associated with TNM stage and therapeutic effect of postoperative chemotherapy in patients with ESCC [17]. Previous studies have indicated that upregulation of TPM3 enhanced the metastatic ability of esophageal cancer cells [18,19]. TPM3 stimulated the epithelial–mesenchymal transition (EMT) process by regulating MMP2 and MMP9 expression, promoting migration of EC cells [20]. However, the reason for the upregulation of TPM3 expression in esophageal squamous cell carcinoma tissues is unclear.

RNA-binding proteins (RBPs) are essential modulators during the post-transcriptional gene regulation [21]. Poly(rC)-binding protein 1 (PCBP1), an RNA-binding protein, is a potential TPM3 mRNA-binding protein. The aim of the current research is to clarify the molecular mechanisms of PCBP1 in upregulating TPM3 expression, providing a potential therapeutic target for the treatment of esophageal squamous cell carcinoma.

## Methods and materials

### Sample collection and clinicopathological features of patients with esophageal squamous cell carcinoma

We retrospectively analyzed the clinical characteristics of 23 patients admitted to our hospital from January 2015 to January 2021 who were diagnosed with esophageal squamous cell carcinoma by gastroscopy and underwent surgical resection, including 14 males and 9 females, aged 52–73 years, with an average of 65.8 ± 7.25 years. Tumors were located in the upper esophagus in 2 cases, in the middle segment in 14 cases, and in the lower esophagus in 7 cases. Seventeen cases were in TNM stage I/II and six cases in stage III/IV. All cases were reconfirmed by postoperative pathology. None of them received antitumor treatments including radiotherapy and chemotherapy before surgery. This study was approved by the ethics committee of Fujian Medical University Union Hospital, and written informed consent was obtained from patients.

## Cell culture

Human esophageal squamous cell carcinoma lines Eca109 and EC1 and human normal esophageal epithelial cells Het-1A were purchased from ATCC (USA) [22]. Het-1A cells were maintained in Bronchial Epithelial Cell Growth Basal Medium (catalog no. CC-3171, Lonza, Basel, Switzerland) supplemented with Bronchial Epithelial Cell Growth Medium BulletKitTM (catalog no. CC-3170, Lonza, Basel, Switzerland). Tumor cells were cultured in DMEM medium containing 10% fetal bovine serum (Gibco), 100 U/ml penicillin, and 100 μg/ml streptomycin (Gibco) in a cell culture incubator with saturated humidity and 5% CO_2_ at 37°C. Culture medium was replaced with fresh medium every 2 days. The cells grew to approximately 90% confluence and then trypsinized using 0.25% Trypsin-EDTA (Gibco) for passaging.

## Plasmid construction and cell transfection

Plasmid pEGFP-N1 was obtained from Public Protein/Plasmid Library (PPL). PCBP1 expression plasmid pEGFP-N1-PCBP1 and TPM3 expression plasmid pEGFP-N1-TPM3 were generated by cloning the coding sequences (CDS) of PCBP1 or the 3′UTR of TPM3 mRNA into pEGFP-N1 vector. The resultant plasmids were transformed into *Escherichia coli* bacterial DH5α. Ten colonies were randomly chosen. The plasmids were extracted and purified according to the instructions of Plasmid Extraction Kit (Sangon Biotech, Shanghai, China) and stored at 4°C.

ESCC cells at logarithmic growth phase were inoculated in 6-well dishes, and cell transfection was performed when the cells grew to approximately 60% confluence using Lipofectamine^™^ 2000 Transfection Reagent (catalog no. 11,668,027, ThermoFisher Scientific). Culture medium was replaced with fresh medium after 12 h, and transfection efficiency was measured by qPCR and western blotting analysis at 48 h post-transfection.

## Generation of PCBP1-knockdown ESCC cell lines

shRNA sequence targeting human PCBP1 (Table 1) was cloned into plv-cs plasmid. The plasmids were transformed into DH5α-competent cells for amplification and purified as described above. For lentiviral generation, 293 T cells were transfected with shRNA plasmids, lentiviral plasmids, and packaging plasmids using Lipofectamine^™^ 2000. Six hours later, culture medium was replaced with fresh medium following continued cultivation for 48 h at 37°C. Next, culture media containing lentiviral particles were harvested, centrifuged at 1000 × g for 10 min at 4°C and then mixed with an equal volume of fresh medium. The mixture was used to infect tumor cells in the presence of polybrene (4 μg/ml final concentration), followed by incubation for 48 h. Infected ESCC cells underwent puromycin selection (2 μg/ml final concentration) for 7 days to obtain ESCC cell lines with stable knockdown endogenous PCBP1. Knockdown efficiency was verified by qPCR and western blotting analysis.

**Table 1.** The sequences of all primers, shRNA and siRNA used in this study

Gene	Forward	Reverse
*GAPDH*	5’-ACAACTTTGGTATCGTGGAAGG-3’	5’-GCCATCACGCCACAGTTTC-3’
*PCBP1*	5’-GACGCCGGTGTGACTGAAA-3’	5’-GTCAGCGTGATGATCCTCTCC-3’
*TPM3*	5’-ACCACCATCGAGGCGGTAA-3’	5’-CCCTTTCCTCCGCATCATCA-3’
**Name**	**siRNA or shRNA sequences**	
shRNA1-PCBP1	5’-ATCTCTTTGATCTTACACCCG-3’	
shRNA2-PCBP1	5’-GGUGUAAGAUCAAAGAGAUCC-3’	
shRNA3-PCBP1	5’-CAUUCCAAAUAACUUAAUUGG-3’	
shRNA-control	5’-GTTCTCCGAACGTG-3’	

## Transwell migration and invasion assays

According to previous work [23], for transwell migration assay, ESCC cell concentration was adjusted to 1 × 10^5^ cells/ml after resuspension with serum-free culture medium. 600 μl of complete culture medium containing 10% FBS was added to transwell lower chamber, and 200 μl of cell suspension in each group was seeded into the upper chamber. After culturing for 24 h, the cells inside the upper chamber were wiped off with wet cotton swabs, and the cells adhering to the lower surface of the polycarbonate membrane were fixed with 4% paraformaldehyde for 15 min, followed by staining with 0.05% crystal violet for 20 min. The stained cells were imaged under an optical microscope, and the number of migrating/invaded cells was counted. Additionally, for transwell invasion assay, the frozen Matrigel (Corning, catalog no. 354248) was thawed overnight at 4°C and diluted to 2 mg/ml using a pre-cooled serum-free medium. 100 μl of diluted Matrigel was added to the upper chamber of the transwell and placed in an incubator at 37°C for 2 h, and the liquid in the upper chamber was gently aspirated before use. All further experimental steps were followed as mentioned above.

## RNA immunoprecipitation (RIP) experiment

RNA immunoprecipitation was performed as described [24]. ESCC cells were collected and lysed with RIP lysis buffer containing RNAase inhibitor and protease inhibitor. Lysates were incubated on ice for 30 min and then centrifuged at 12,000 × g for 10 min at 4°C. The supernatant was collected for subsequent analysis. The samples were incubated with the corresponding antibodies or protein A/G-coupled magnetic beads that were washed three times beforehand with appropriate amount of RIP lysis buffer, following incubation with rotating overnight at 4°C. After carefully aspirating off the supernatant to collect the beads, the beads were washed three times with washing buffer for precipitation. Total RNA in the precipitate was extracted using RNA Isolation Kit (catalog no. 83913, Sigma-Aldrich) and prepared for subsequent experiments.

## Quantitative polymerase chain reaction (qPCR) analysis

Total RNA was extracted and purified from ESCC cells or tissues using RNA Isolation Kit (Sigma-Aldrich), and the purity and concentration of total RNA were measured using NanoDrop (ThermoFisher Scientific). cDNA was synthesized using PrimeScript™ RT Master Mix (Perfect Real Time) (catalog no. RR036A, Takara) following the manufacturer’s instructions. 2 μl of reverse transcription reaction product was used as qPCR reaction template. qPCR analysis was performed using One-Step TB Green® PrimeScript™ RT-PCR Kit (Perfect Real Time) (catalog no. RR066A, Takara). Total reaction system was 20 μl: 2 μl reverse transcription product, 10 μl 2× Mix, 0.8 μl each forward/reverse primers, and 6.4 μl ddH_2_O. qPCR thermal cycling conditions were as follows: pre-denaturation for 3 min at 95°C, followed by 40 cycles of denaturation at 95°C for 30 s, annealing at 60°C for 30 s, and extension at 72°C for 10 s. Relative gene expressions were calculated using the 2^−ΔΔCt^ method using GAPDH as reference gene. Primer sequences are listed in Table 1.

## Western blotting

Tissue and cell samples were lysed using pre-chilled RIPA lysis buffer containing 1% PMSF. After incubating on ice for 10 min, the suspension was centrifuged at 12,000 × g for 15 min at 4°C. The supernatant containing proteins was collected, and protein concentration was determined using Pierce™ BCA Protein Assay Kit (catalog no. 23225, ThermoFisher Scientific). Next, 30 μg of heat-denatured protein lysates were loaded onto SDS–PAGE gels for electrophoretic separation. The separated proteins were transferred to PVDF membranes by a wet-transfer method, and the membranes were blocked with 5% skimmed milk in TBST for 2 h at room temperature. Subsequently, the membranes were incubated with the corresponding antibodies overnight at 4°C. After washing three times with TBST, the secondary antibodies were added and incubated for 2 h at room temperature. The membranes were again washed three times with TBST, and immunoblots were visualized using ECL chemiluminescence kits (Pierce). Protein expression was quantified by analyzing grayscale values of bands using Quantity One software with β-actin as the internal reference.

## Immunohistochemistry (IHC) of PCBP1 and TPM3 in ESCC tissues

Tissue paraffin sections were obtained from the Department of Pathology. The sections were processed by standard experimental procedures, and the primary antibodies used were as follows: Anti-PCBP1 (Proteintech Group, Inc., #14523-1-AP, dilution 1:200) and anti-TPM3 (Proteintech Group, Inc., #10737-1-AP, dilution 1:50).

## Bioinformatic analysis

Gene expression between esophageal cancer specimens and normal tissues and correlation between gene expressions were further analyzed using GEPIA (http://gepia.cancer-pku.cn/), TCGA, and GTEx datasets.

## Statistical analysis

Data were analyzed using SPSS 21.0 software and expressed as mean ± standard deviation. Comparisons between two groups were performed with independent sample *t*-test or paired student *t*-test. One-way ANOVA with Dunnett post hoc test was used for multiple comparisons. The Kaplan–Meier method and the log-rank test were used to estimate the overall survival (OS). Pearson correlation analysis was applied to assess the correlation of gene expressions. A *P* value less than 0.05 was considered to be statistically significant.

## Results

Previously, we found the abnormally high expression of TPM3 in ESCC tissues compared to normal tissues [17]; however, the mechanism underlying the upregulation of TPM3 expression is unclear. RNA-binding proteins (RBPs) are important regulators of RNA metabolism by interacting with RNA [25,26]. PCBP1, a well-characterized RNA-binding protein, was identified as the RNA-binding protein to TPM3 mRNA. Therefore, to understand the biological functions of PCBP1 in esophageal squamous cell carcinoma, qPCR, western blotting assays, and cellular functional experiments were performed. Here, we found that PCBP1 was highly expressed in esophageal squamous cell carcinoma and PCBP1 knockdown suppressed ESCC cell migration and invasion, suggesting that PCBP1 is an ESCC-associated pro-oncogenic factor.

## PCBP1 is highly expressed in esophageal cancer tissues and ESCC cells

**Figure 1.**
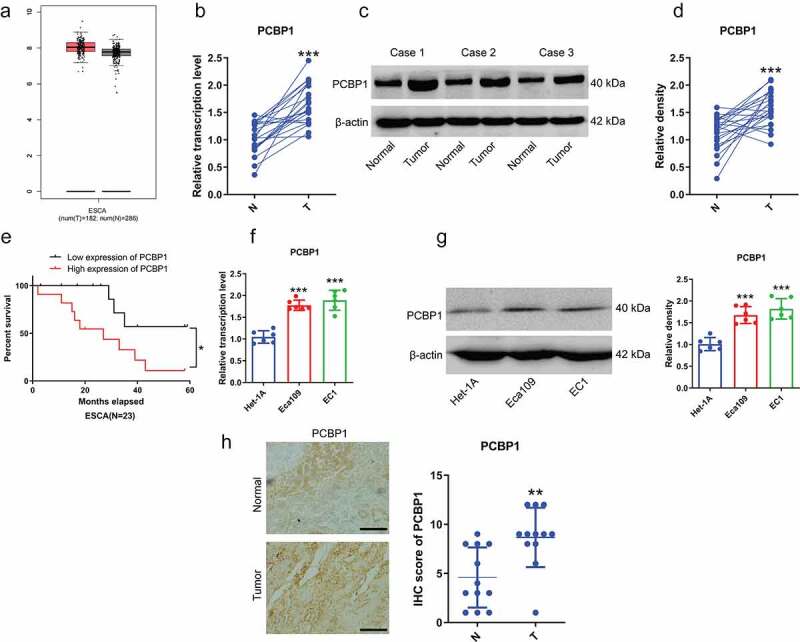
PCBP1 expression in esophageal squamous cell carcinoma tissues and cells. (a) PCBP1 expression in esophageal squamous cell carcinoma from GEPIA dataset. (b) PCBP1 mRNA expression in 23 paired esophageal squamous cell carcinoma tissues and normal tissues. (c, d) Representative images and quantification of PCBP1 protein expression in esophageal squamous cell carcinoma tissues and adjacent normal tissues. (e) Correlation between PCBP1 expression and ESCC patients’ prognosis. (f) PCBP1 mRNA expression in ESCC cell lines and normal esophageal epithelial cells Het-1A. (g) Representative blots and statistical analysis of PCBP1 protein expression in ESCC cells and Het-1A cells. (h) Immunohistochemistry of PCBP1 in ESCC tissues. Comparisons between two groups were performed with paired student *t*-test. One-way ANOVA with Dunnett post hoc test was used for multiple comparisons. Kaplan–Meier method and the log-rank test were used to estimate overall survival (OS). Error bars represented as S.D. *P < 0.05; **P < 0.01; ***P < 0.001. ESCC, esophageal squamous cell carcinoma.

Our data indicated that PCBP1 was significantly upregulated in ESCC tissues, which was consistent with the data from TCGA database (Figure 1(a-d)). ESCC patients with high expression of PCBP1 had shorter survival time (Figure 1(e)). Besides, PCBP1 was significantly elevated in ESCC cell lines compared to human normal esophageal epithelial cells (Figure 1(f,g)). Besides, immunohistochemistry revealed that the IHC scores of PCBP1 in ESCC tissues were markedly higher than adjacent normal tissues (Figure 1(h)).

## PCBP1 knockdown reduces migratory and invasive capabilities of ESCC cells

**Figure 2.**
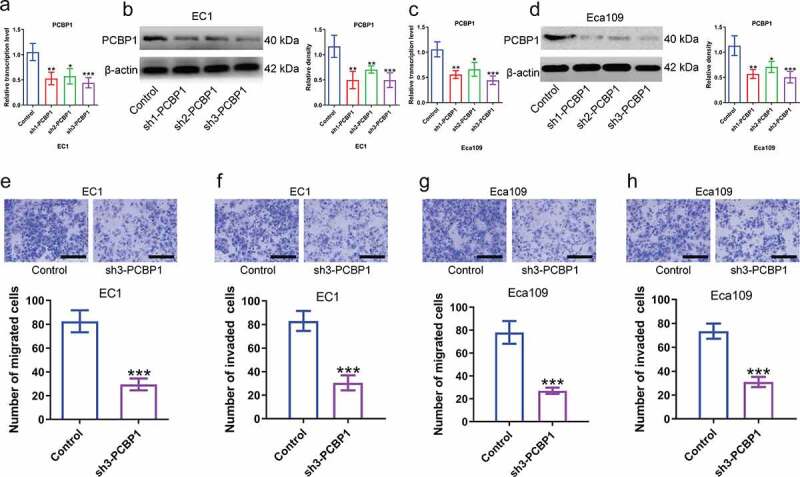
Effects of PCBP1 knockdown on migration and invasion of esophageal squamous cell carcinoma cells. (a, b) Knockdown efficiency of three shRNAs targeting PCBP1 in EC1 cells was tested by qPCR and western blot analysis. (c, d) Knockdown efficiency of three shRNAs targeting PCBP1 in Eca109 cells was measured by qPCR and western blot analysis. (e, f) PCBP1 knockdown attenuated migratory and invasive abilities of EC1 cell. (g, h) PCBP1 knockdown reduced migratory and invasive capabilities of Eca109 cell. Comparisons between two groups were performed with unpaired student *t*-test. One-way ANOVA with Dunnett post hoc test was used for multiple comparisons. Error bars represented as S.D. *P < 0.05; **P < 0.01; ***P < 0.001.

Next, to explore the biological function of PCBP1 in esophageal squamous cell carcinoma, we constructed ESCC cell lines with stable PCBP1 knockdown and examined the silencing efficiency by qPCR and western blotting analysis (Figure 2(a-d)). Data indicated that PCBP1 knockdown could significantly inhibit the migration and invasion of ESCC cells compared to the control cells. The inhibitory effects of different shRNAs targeting PCBP1 on ESCC cell migration and invasion were correlated with PCBP1 expression levels (Figure 2(e-h)).

## PCBP1 directly binds to the 3’UTR of TPM3 mRNA and regulates TPM3 mRNA stability

**Figure 3.**
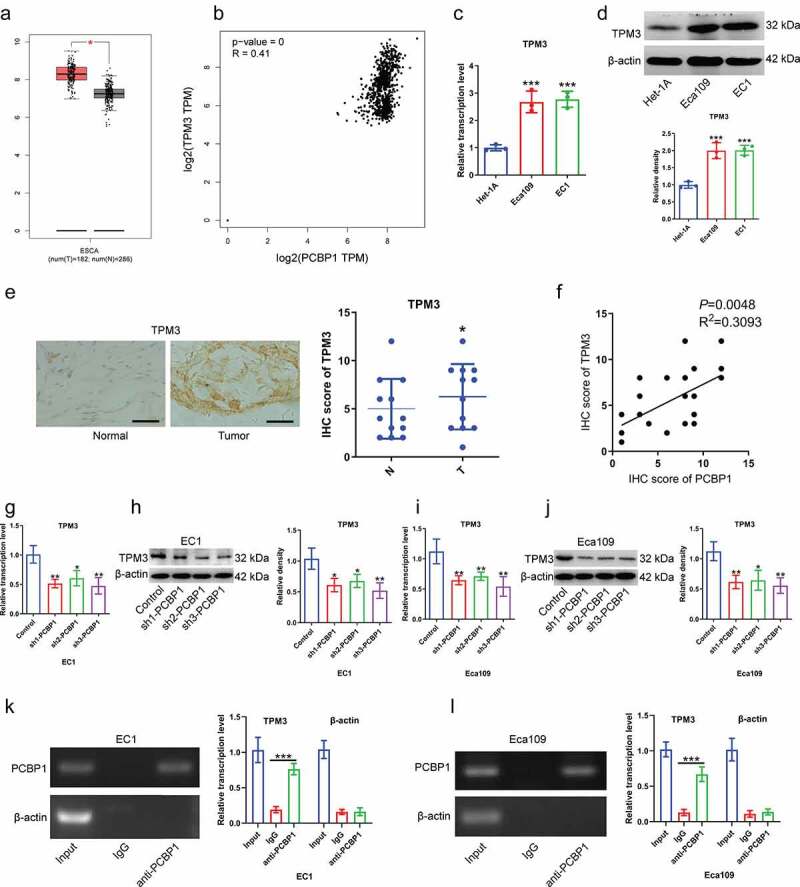
TPM3 is a downstream target of PCBP1 in esophageal squamous cell carcinoma cells. (a) TPM3 expression from TCGA esophageal squamous cell carcinoma dataset. (b) Correlation between TPM3 and PCBP1 expression from TCGA esophageal squamous cell carcinoma dataset. (c, d) TPM3 mRNA and protein levels in esophageal cancer cell lines and normal esophageal epithelial cells Het-1A. (e) Immunohistochemistry of TPM3 in ESCC tissues. (f) Correlation between the IHC scores of PCBP1 and TPM3 in ESCC tissues. (g, h) PCBP1 knockdown caused a significant decrease in TPM3 expression at the transcriptional and translational levels in EC1 cells. (i, j) PCBP1 knockdown resulted in a significant downregulation in TPM3 expression at the transcriptional and translational levels in Eca109 cells. (k, l) RIP assay showing an interaction between TPM3 and PCBP1 in EC1 and Eca109 cells. Comparisons between two groups were performed with unpaired student *t*-test. One-way ANOVA with Dunnett post hoc test was used for multiple comparisons. Error bars represented as S.D. *P < 0.05; **P < 0.01; ***P < 0.001.

TPM3 was highly expressed in esophageal squamous cell carcinoma from TCGA dataset (Figure 3(a)). Data from GEPIA dataset showed a significant positive correlation between PCBP1 and TPM3 expression (Figure 3(b)). Besides, TPM3 expression was significantly elevated in ESCC cells compared to normal esophageal epithelial cells (Figure 3(c,d)). Immunohistochemistry demonstrated that the IHC scores of TPM3 were significantly elevated in ESCC tissues compared with in adjacent normal tissues (Figure 3(e)), and TPM3 expression was positively correlated with PCBP1 expression in ESCC tissues (Figure 3(f)). Considering the difference in knockdown efficiency of shRNAs targeting PCBP1, we thus chose shRNA 3 (sh3) with the highest silencing efficiency for subsequent experiments. PCBP1 knockdown caused downregulation of TPM3 mRNA and protein levels in ESCC cells (Figure 3(g-j)).

**Figure 4.**
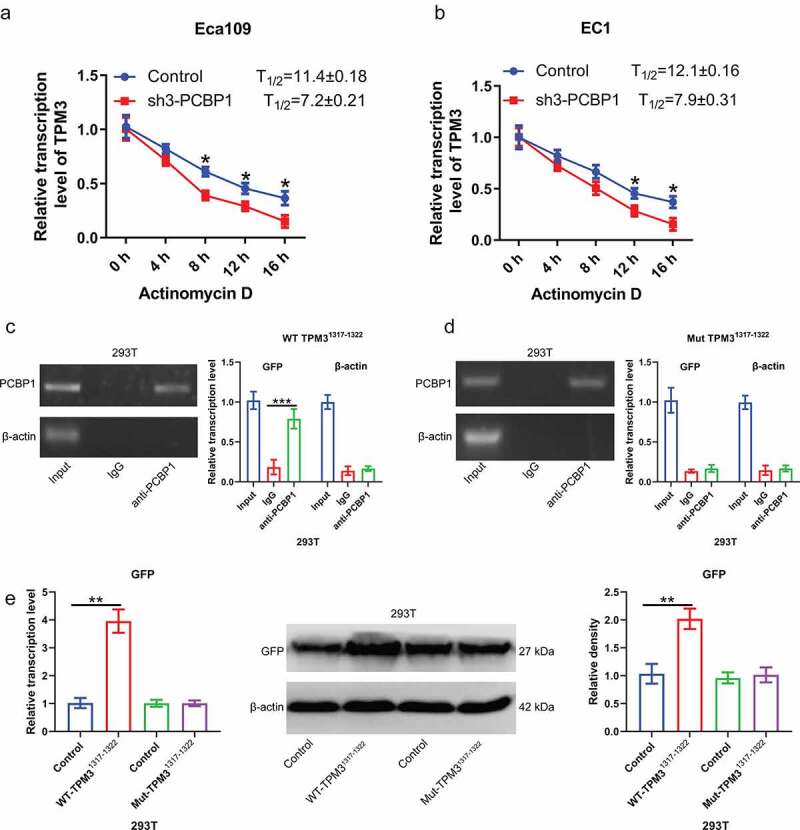
PCBP1 maintains TPM3 mRNA stability by directly binding to 3’UTR of TPM3 mRNA. (a, b) TPM3 mRNA levels in EC1 cells and Eca109 cells with PCBP1 knockdown. The cells were treated with actinomycin D and harvested at the indicated times. (c) RIP analysis showing the interaction between wild type TPM3^1317−1322^ mRNA transcripts and PCBP1. (d) RIP analysis showing the interaction between mutant TPM3^1317−1322^ mRNA transcripts and PCBP1. (e) qPCR and western blot analysis were performed to assess GFP protein expression in 293 T cells transfected with TPM3^1317−1322^-wt plasmid or TPM3^1317−1322^-mut plasmid. Comparisons between two groups were performed with unpaired student *t*-test. Error bars represented as S.D. *P < 0.05; **P < 0.01; ***P < 0.001. WT, wild type; Mut, mutant.

To determine the relationship between PCBP1 and TPM3, RIP experiments were conducted. Result confirmed that anti-PCBP1 magnetic beads were able to enrich TPM3 mRNA but not β-actin mRNA, suggesting that PCBP1 directly binds to TPM3 mRNA (Figure 3(k,l)). Moreover, TPM3 mRNA content in actinomycin D-treated EC cells was measured. It was observed that the degradation rate of TPM3 mRNA was faster after PCBP1 knockdown in comparison to the control (Figure 4(a,b)).

Given that PCBP1 directly binds to the GCCCAG motif of RNA, we then cloned the sequence at 1317–1322 of TPM3 mRNA 3’UTR into pcDNA3.1-GFP plasmid and then co-transfected with PCBP1 overexpression plasmid into 293 T cells. Anti-PCBP1 magnetic beads were able to enrich GFP mRNA; however, after mutating the binding sites of TPM3 mRNA, anti-PCBP1 magnetic beads were unable to enrich GFP mRNA, suggesting that PCBP1 exerted its biological activity by directly binding to TPM3 mRNA 3’UTR (Figure 4(c,d)). Wild-type TPM3^1317−1322^ elevated GFP protein expression, while mutant TPM3^1317−1322^ had no significant effect on the GFP expression (Figure 4(e)).

## PCBP1 regulates TPM3 expression, affecting migration and invasion of ESCC cells

**Figure 5.**
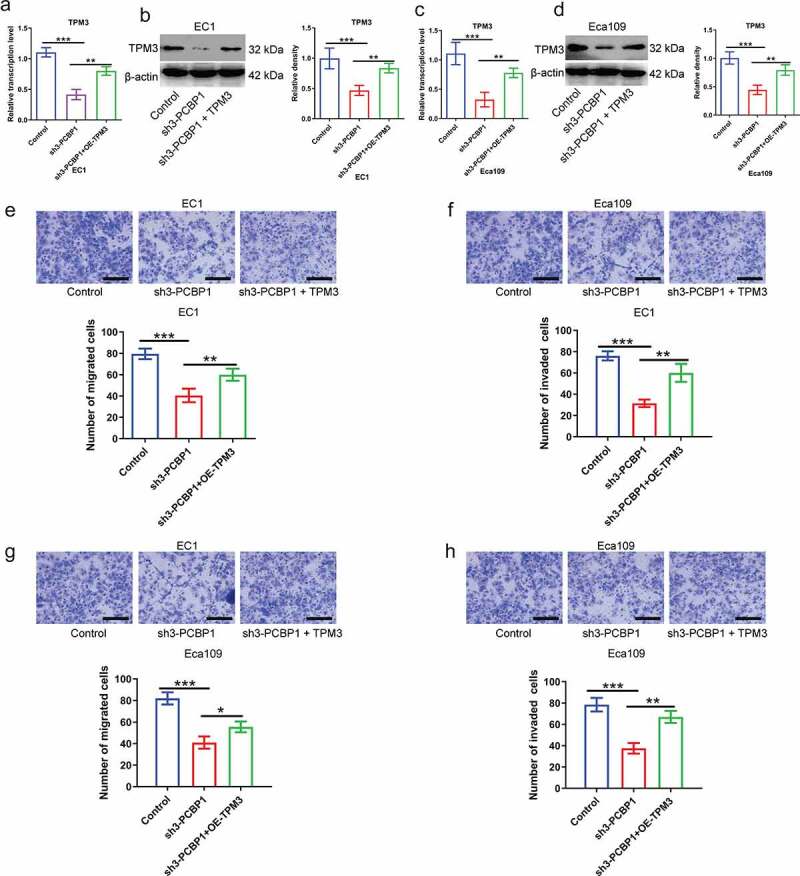
PCBP1 directly regulates TPM3 expression affecting migration and invasion of esophageal squamous cell carcinoma cells. (a, b) The mRNA and protein levels of TPM3 in PCBP1-knockdown EC1 cells transfected with or without TPM3 overexpression plasmids. (c, d) The mRNA and protein expressions of TPM3 in PCBP1-knockdown Eca109 cells transfected with or without TPM3 overexpression plasmids. (e, f) TPM3 overexpression partially reversed migration and invasion of EC1 cells induced by PCBP1 knockdown. (g, h) TPM3 overexpression partially restored migration and invasion of Eca109 cells caused by PCBP1 knockdown. Data were analyzed using one-way ANOVA with Dunnett post hoc test. Error bars represented as S.D. *P < 0.05; **P < 0.01; ***P < 0.001.

To further examine the effect of PCBP1/TPM3 axis on malignant phenotypes of esophageal cancer cells, plasmid-encoded TPM3 restored TPM3 expression in PCBP1-knockdown ESCC cells (Figure 5(a-d)). PCBP1 knockdown attenuated the migratory and invasive abilities of ESCC cells, whereas overexpression of TPM3 partially reversed the phenotypes induced by PCBP1 knockdown compared to the control (Figure 5(e-h)). These findings suggested that the PCBP1/TPM3 axis did affect the migratory and invasive properties of esophageal squamous cell carcinoma cells.

## Discussion

TPM3 is a cell motility-related protein that is upregulated in a variety of tumor tissues and promotes tumor cell migration and invasion [27]. Similarly, our previous study showed that TPM3 was upregulated in esophageal squamous cell carcinoma tissues and was associated with migration and invasiveness of ESCC cells [17]. Therefore, we further explored the molecular mechanisms underlying the upregulation of TPM3 in esophageal squamous cell carcinoma. In this study, we identified PCBP1 as an upstream regulator of TPM3. PCBP1 is an RNA-binding protein that was aberrantly expressed in a variety of tumor tissues [^28–31^]. In prostate and breast cancers, PCBP1 expression was significantly upregulated and correlated with poor outcomes, and PCBP1 acts as a pro-oncogenic factor [28,29]. In the current study, we found that PCBP1 was responsible for the significant upregulation of TPM3 in esophageal squamous cell carcinoma. PCBP1 is an RNA-binding protein (RBP). PCBP1 was highly expressed in esophageal cancer tissues. PCBP1 knockdown significantly attenuated the proliferative, migratory, and invasive capabilities of ESCC cells by directly binding to the 3′ untranslated region (3’UTR) of TPM3 mRNA and regulating TPM3 mRNA stability. Targeting PCBP1 holds great promise for treating esophageal squamous cell carcinoma.

RNA-binding proteins (RBPs) play a pivotal role in a variety of biological processes, especially in the post-transcriptional gene regulation [32]. Considering that PCBP1 is an RNA-binding protein, previous studies have shown that PCBP1 directly binds to mRNA, regulating mRNA stability [29,33]. There was a significant positive correlation between PCBP1 and TPM3 expression in ESCC. Therefore, we hypothesized that PCBP1 could directly bind to TPM3 mRNA and had an effect on mRNA stability. Our results confirmed that PCBP1 could directly bind to TPM3 mRNA at position 1317–1322 to stabilize mRNA against degradation. PCBP1 knockdown simultaneously accompanied a decrease in TPM3 expression and inhibited migration and invasion of ESCC cells. Although TPM3 plays a complex role in a variety of human tumors [18,34], to our knowledge, this is the first study to confirm post-transcriptional regulation of TPM3 mRNA mediated by RNA-binding proteins in esophageal squamous cell carcinoma.

RBPs bind to RNA-forming ribonucleoprotein (RNP) complexes and contribute to RNA maturation, translation, transportation, and localization [32]. RBP consists of multiple sub-domains that can efficiently recognize a broad range of target RNAs [26]. Via eCLIP-sequence analysis to identify PCBP1-interacting transcripts in hepatocellular carcinoma cells, it was found that PCBP1 reads were preferentially enriched in 3′UTR and CDS regions. Notably, a total of 145 genes that were identified to bind to PCBP1 underwent alternative splicing under the regulation of PCBP1 at the same time [35]. Other works in various human cancer cells uncovered that PCBP1 binds to p27 mRNA 3’-UTR region via 5’-AUUAAGUAAU-3’ to repress tumor cell transformation and carcinogenesis [29]. In ovarian cancer, RNA-affinity pulldown using biotinylated TRIM56 3’-UTR combined with mass spectrometry has found PCBP1 as the most differentially binding protein. Overexpression of PCBP1 promoted the migration and invasion of ovarian cancer cell SKOV-3 [36]. Hence, it is suggested that PCBP1 knockdown-caused alterations in cellular behaviors related to metastatic ability may be a part of the mechanisms by which it performs its biological function in ESCC. Further investigations are required to understand the underlying mechanisms of PCBP1 in ESCC progression.

## Conclusions

In summary, our data suggested that PCBP1 expression levels were significantly increased in esophageal squamous cell carcinoma. Upregulated PCBP1 led to significant upregulation of TPM3 in cancer tissues by directly binding to TPM3 mRNA and enhancing its mRNA stability, promoting migration and invasion of ESCC cells. These findings provide a new perspective for understanding molecular mechanisms of esophageal carcinogenesis. PCBP1 may be a potential therapeutic target for esophageal squamous cell carcinoma treatment.

## Data Availability

The authors confirm that the data supporting the findings of this study are available within the article [and/or] its supplementary materials.
